# Senile Decay

**Published:** 1884-12

**Authors:** J. Smith Dodge


### SENILE DECAY.

BY J. SMITH DODGE, JR., M. D., D. D. S.

Every dentist witnesses the rapid decay of teeth in persons of advanced age, and the phrase " senile decay " is an accepted one. Yet little or nothing is found in the books about this condition.

Of course, in general the causes and characteristics of decay are the same at all times of life, but in the very old the process has peculiarities of its own, which have both scientific and practical interest. At what age we may be called *old*, and therefore entitled to lose our teeth in this way, cannot of course be stated, but my observation has found the peculiarities about to be described only in those who exhibit signs of general bodily decay; and we may perhaps fix the period in which senile decay actually comes under the dentist's hands at from sixty-five to eighty.

The teeth thus attacked are naturally such as have resisted the wear and tear of a lifetime. And I have generally been struck with a certain perfection of form and color, a good, wholesome look, which would put them in the very highest class of teeth. I think, too, I have oftenest seen this decay in teeth never previously attacked, although that is not constant. The great and striking feature is what may be called the *unbounded* character of the decay. With a moderate opening the whole interior of the crown may be destroyed, leaving little more than the shell of enamel. At the cervical border, too, proximal cavities extend under the gum to a most unusual degree. Another peculiarity is the rapidity with which the destruction proceeds. As these patients are seldom accustomed to have regular periodic examinations, it is not always easy to judge how long the trouble may have existed. But I have more than once seen senile decay far advanced in teeth which had only a few months before been examined and found good. Rapid progress, too, is proved by the large amount of decalcified dentine not yet softened, which is one of the constant features of this condition. Still more noticeable is the dulled sensibility of these teeth. I have hardly ever heard of their aching, and the process of excavating gives little or no pain. Indeed, care must be used, or a drop of blood will be the first indication that the pulp is in danger. I have several times cut to the pulp, so that it bled, without the patient's feeling it.

These characteristics all seem to be dependent on a greatly reduced innervation of the tooth. Many facts might be appealed to, some familiar and some less observed, to prove that the great nervous supply of the teeth is needed to maintain their functional competence. And when the tooth is attacked by decay, all this power is used to resist the disease. In favorable cases it is certain that a protective zone surrounds the decayed dentine, limiting, retarding, or even arresting its progress. In all cases it is probable that the effort is made, and has some result, so long as the activity of the dental nerves remains unimpaired. Of course the heightened sensibility of carious cavities is the result of this intensified innervation. But when the general decline of vitality reaches the dental nerves, they are no longer able to respond to local attacks. All resistance to destructive agencies is at an end, and the unlimited, rapid, painless decay above described, takes place. The differences, therefore, between senile decay and the decay of earlier life should furnish valuable hints for the study of that vital resistance to decay which is as yet an unregarded, but a vastly important factor in its history.

The chief practical interest of senile decay attaches to the treatment. The use of gold is almost out of the question. Aged patients cannot and should not be asked to bear the strain of very tedious dentistry. And from the analogy of the rapidly decaying teeth of children it may be doubted whether gold, if used, would preserve the tooth. Amalgam has not given me satisfaction. The enamel soon fails about the filling, and the same process is resumed, with the same speedy results. But gutta percha meets the case exactly. The mastication upon such teeth is not forcible, and the softness of the material hardly counts here, while its protective power is exactly what these teeth call for. I have again and again seen the mere outline of a tooth filled with gutta percha hold its own beyond all expectation, to the great pleasure and advantage of its owner. In fact, the *remnant* of a gutta-percha filling in such a tooth is worth all the metal it can be made to hold. The edges of enamel may stand out, the soft filling may have been grooved toward the grinding surface and massed into a projection against the cervical wall, the surface may have grown brown and unsightly, but the old man will assure you his tooth is a daily comfort, and it would be a sad blunder indeed to take out the unsightly plug and make thorough work of it with metal. "Handsome is that handsome does."

